# Experiences of positive encounters with healthcare professionals among women on long-term sickness absence due to breast cancer or due to other diagnoses: a nationwide survey

**DOI:** 10.1186/s12889-019-6666-8

**Published:** 2019-03-29

**Authors:** M. Söderman, A. Wennman-Larsen, K. Alexanderson, E. Friberg

**Affiliations:** 1 Division of Insurance Medicine, Department of Clinical Neuroscience, Karolinska Institutet, SE-171 77 Stockholm, Sweden; 2 Sophiahemmet University, Stockholm, Sweden

**Keywords:** Breast cancer, Sick leave, Encounter, Return to work

## Abstract

**Background:**

Experiences of encounters with professionals have been shown to influence return to work (RTW) among sickness absentees in general. The aim was to gain knowledge on experiences of encounters with healthcare professionals and the ability to RTW among women on long-term sickness absence (SA) due to breast cancer (BC) compared to among women on long-term SA due to other diagnoses.

**Methods:**

Analyses of questionnaire data about experiences of encounters with healthcare professionals among 6197 women aged 19–65 years and on a SA spell lasting 4–8 months. Of those, 187 were on SA due to BC. Descriptive statistics and adjusted (for age, birth country, educational level, depressive symptoms) logistic regression analyses with 95% confidence intervals (CI) were conducted.

**Results:**

About 95% in both groups of women stated that they had experienced positive encounters with healthcare, and a minority, about 20%, had experienced negative encounters. Four specific types of positive encounters had been experienced to a lesser extent by women with BC: “allowed me to take own responsibility” (odds ratio (OR) 0.6; 95% CI 0.4–0.8), “encouraged me to carry through my own solutions” (OR 0.5; 95% CI 0.4–0.7), “made reasonably high demands” (OR 0.6; 95% CI 0.4–0.9), and “sided with me/stood on my side” (OR 0.6; 95% CI 0.4–0.8). Among the women with BC, 46% stated that positive encounters promoted their ability to RTW compared to 56% among the others. Conclusion: Most of the women had experienced positive encounters and about half stated that positive encounters promoted their ability to RTW, although a slightly smaller proportion of the women with BC stated that. This study emphasizes that not only medical treatment but also encounters may influence the ability to RTW, something that is of clinical importance.

## Background

Globally, breast cancer (BC) is the most common type of malignancy among women, with an increasing survival rate [1]. In Sweden, around 9700 women are diagnosed with BC every year with a five-year survival rate of 90% [2] and a majority (66%) of these women are of working ages [3]. Cancer survivors in general [4] as well as those diagnosed with BC [5] have reported that return to work (RTW) is an important part of their recovery. During the first year after diagnosis, most women with BC have been observed to be on sickness absence (SA) at least for some time [6, 7] and the probability of RTW among women with BC after the first and up to three years after diagnosis varies largely between countries; from 43% up to 93% [6–11]. So far, most studies on SA have focused on risk factors for becoming sickness absent [7, 11, 12]. However, in clinical settings, knowledge is also warranted on factors that are associated with RTW among patients who are already sickness absent. There are a number of factors, at different structural levels, that have been shown to be associated with RTW among sickness absentees in general [13]. One such possible factor is sickness absentees’ experiences of encounters with healthcare professionals [14–26]. Yet, the research on how sickness absentees experience encounters with healthcare professionals in relation to RTW is limited [19–23, 25, 26]. Previous studies of patients on long-term SA have shown that negative encounters from healthcare professionals can make patients feel wronged [16, 23] or influence their RTW [27]. Other studies observed that positive encounters in particular promoted the ability to RTW among sickness absentees [19, 22, 26, 28]. Furthermore, some studies have focused on specific types of positive and negative encounters during long-term SA [15, 29]. Sickness absentees’ encounters with healthcare professionals that are characterized by professionalism, knowledge, continuity, and a holistic approach have been observed to create trust [28]. Most previous studies have studied encounters with healthcare among people on SA in general [16, 18, 19, 21–26], irrespective of SA diagnosis. One exception from this is studies of people on SA due to heart-failure diagnosis [27]. However, no previous study has focused on whether experiences of women on long-term SA due to BC are in line with those of other women on SA. Such knowledge is needed as base for interventions specific for women with BC.

In previous studies of SA [13] and also in studies of how women on SA experience encounters with healthcare professionals, associations with age, educational level, and birth country have been observed [18, 19, 24, 25, 27]. Regarding previous studies of BC, studies on factors associated with RTW show similar results as those for sickness absentees in general regarding sociodemographic factors [7, 12]. Few studies have, however, been conducted within this research field of BC concerning encounters from healthcare [8, 30–34]. In Sweden, the care and treatment of women with BC are outlined in scientifically based National Guidelines [3], including recommendations of the use of multi-professional teams for each woman in order to get consensus for diagnosis, treatment, and follow-up. These National Guidelines also include information about the responsibility of the contact nurse to inform patients about the impact of the disease and treatment on work capacity and SA. As mentioned above, previous research shows that experiences of encounters may influence RTW among sickness absentees in general. However, knowledge is lacking regarding experience of encounters among women with BC and especially on whether this influences their RTW. The experiences among women on SA due to BC might also differ from women on SA due to other diagnoses.

## Methods

The aim was to gain knowledge on the experiences of encounters with healthcare professionals and the ability to RTW among women on long-term SA due to BC compared with women on long-term SA due to other diagnoses.

In April 2013, a questionnaire in Swedish was sent to a random sample of 17,395 of all individuals in Sweden who had an ongoing SA spell that had lasted at least 4, and not more than 8 months; about half of the population with such SA spells in Sweden at that time. The participants were identified by the Swedish Social Insurance Agency. All people from 16 years of age with a minimum level of income from work, unemployment benefits, or parental benefit who due to disease or injury have reduced work capacity can be granted SA benefits by the Social Insurance Agency. After the seventh day of a SA spell, a certificate from a physician is required.

The comprehensive questionnaire included many questions about experiences of positive and negative encounters with healthcare professionals as well as with Social Insurance Agency officers. It was a slightly revised version of a previous questionnaire that was based on empirical and theoretical studies [19, 29, 35, 36]. The questionnaire was mailed to the participants’ home addresses by Statistics Sweden, who also linked register data to each individual, using the unique personal identity number assigned to all people living in Sweden. Such data were obtained from two authorities: Statistics Sweden (regarding sociodemographic factors e.g., age, country of birth, educational level) and the Social Insurance Agency (regarding SA diagnoses). The research group thereafter received the anonymized data.

For the analysis, age was categorized in three groups (19–44, 45–54, 55–65), and for the logistic regression analyses in two groups (19–54, 55–65), country of birth dichotomized as born in Sweden or elsewhere, educational level dichotomized as primary/secondary school (≤12 years) or college/university (> 12 years).

In this study, answers to questions regarding encounters with healthcare professionals were analyzed. Women on SA due to BC were compared to women with other SA diagnoses regarding their experiences of positive or negative encounters with healthcare professionals during the current SA spell, as well as regarding specific such encounters.

A question about the SA diagnosis was asked: “With what diagnosis/disorder are you sickness certified?”, with three response alternatives (pain or aching in muscles or joints, mental disorders, and other – for the latter the participant was asked to specify). Two general questions about positive and negative encounters were asked; “Did you experience a positive encounter with someone in healthcare during your sickness absence?” and “Did you experience a negative encounter with someone in healthcare during your sickness absence?” with response alternatives “yes or no”. In addition, the questionnaire included 19 statements about specific types of positive encounters with a healthcare professional during the SA spell (presented in Table 2) and 25 statements about specific types of negative encounters (not presented as explained in data analysis), with four response alternatives (agree completely, agree to some extent, disagree to some extent, and disagree completely). Participants who answered yes to either the general question or to any of the questions about specific encounters were considered as having experienced any positive or negative encounters, respectively. Also, two questions about whether the positive or negative encounters were perceived to have influenced the ability to RTW were included; “Have positive encounters from healthcare influenced your ability to return to work?” and “Have negative encounters from healthcare influenced your ability to return to work?”, with six response alternatives (hindered to a great extent, hindered to some extent, no influence, promoted to some extent, promoted to a great extent, and not had any positive/ negative encounter (not included in analyses)). These were categorized as “hindered”, “had no influence”, or “promoted” for the analyses. Moreover, the questionnaire included a question on whether participants’ contacts with different healthcare professions (physicians, registered nurses, physiotherapists, clinical social worker/psychologists, occupational therapists, and naprapath/chiropractor) most often had been positive or negative; “Are your contacts with the following professions most often positive or negative?” on a five-degree scale (very positive, quite positive, quite negative, very negative, and not had contact). The answers were dichotomized as “most often positive” (very positive, quite positive) or “most often negative” (quite negative, very negative)" when analyzed. The answer “not had contact” was not included in analyses. Depressive symptoms are not unusual among individuals on SA and may influence how respondents perceive and answer questions [37, 38]. To account for this, we used two questions to assess self-rated depressive symptoms. Participants were asked whether in the last 12 months they had felt low and/or had a lower interest for activities during the larger part of the day for at least two weeks, and if yes, whether this had been the case for the last two weeks. Participants who responded yes to both questions were categorized as having depressive symptoms.

### Study sample

From the random sample of 17,395 sickness absentees, 11,288 (64.9%) were women, and of these women a total of 6254 answered the questionnaire (response rate among women 55.4%). In most surveys, the response rate is lower among those with lower education, of lower ages, and among those born in another country [39, 40]; this was also the case here. After excluding 15 women due to missing data on the general questions about encounters with healthcare and those older than 65 years old at the end of the year, a total of 6197 women aged 19–65 years (i.e., “ordinary” working age in Sweden) remained for analyses. Of them, 187 women either had BC stated on the medical certificate as the main SA diagnosis (*n* = 157) or reported in the questionnaire that they were on SA due to BC (*n* = 30). These 187 women were compared to the remaining 6010 women with other SA diagnoses. According to the SA certificates of those 6010 women 2228 (37%) were on SA due to mental diagnoses, 1775 (30%) due to musculoskeletal diagnoses, 373 (6%) due to injuries, 219 (4%) due to other cancer diagnoses, 158 (3%) due to circulatory diagnoses, and 373 (6%) due to other diagnoses.

### Data analysis

Descriptive statistics were calculated for the characteristics of the study population, encounters with healthcare professionals, and specific types of encounters. Chi^2^-tests were used to determine statistical differences between groups. In the answers to the initial general questions about experiences of encounters, it was observed that most women had experienced positive encounters, while few had experienced negative encounters. Also, the contacts with healthcare professionals were most often positive. Therefore, odds ratios (OR) with 95% confidence intervals (CI) were calculated with logistic regression to compare groups regarding their experiences of positive encounters and if positive encounters had influenced their RTW. Both crude analyses and analyses adjusted for age, country of birth, educational level, and depressive symptoms were conducted. In sensitivity analyses the women with SA due to other cancer diagnoses where excluded from the comparison group. SAS 9.4 and SPSS 24 were used for the analyses.

## Results

The 187 women on SA due to BC were somewhat older than the 6010 women with other SA diagnoses (mean age BC 52.6, mean age other 47.9, *p* = 0.02). In both groups, the majorities were born in Sweden (BC 84.0%, other 86.4%, n.s.) and about half had primary or secondary school as their highest educational level (BC 50.3%, other 57.0%, n.s.) (Table 1). A smaller proportion of the women with BC reported depressive symptoms compared to among the women with other SA diagnoses (BC 23.5%, other 32.5%, *p* = 0.01).

**Table 1:** Characteristics of women on long-term sickness absence (SA) due to breast cancer (BC) or due to other diagnoses; frequencies, percent, and *p*-values

	Women on SA due to BC	Women on SA due to other diagnoses	
	*n* = 187	*n* = 6010	
	n (%)	n (%)	p
Age^a^			0.02^a^
19–44	31 (16.6)	2233 (37.2)	
45–54	75 (40.1)	1658 (27.6)	
55–65	81 (43.3)	2119 (35.3)	
Country of birth			
Sweden	157 (84.0)	5195 (86.4)	
Elsewhere	30 (16.0)	815 (13.6)	0.33
Educational level			
Primary/secondary school	94 (50.3)	3424 (57.0)	
College/university	93 (49.7)	2586 (43.0)	0.07
Depressive symptoms			
No	143 (76.5)	4060 (67.5)	
Yes	44 (23.5)	1950 (32.5)	0.01

^a^*p*-value for the continuous variable

### Specific types of experienced encounters

Distributions and ORs of answers regarding specific types of encounters in the two groups of women are presented in Table 2. Similar proportions of women in the two groups had experienced any positive encounter (95% in both groups) or any negative encounter (BC 18.7%, other 23.6%) with healthcare. In general, a high proportion of women in both groups also reported that they had experienced the specific types of positive encounters. There were, however, significant differences between women with BC and women with other SA diagnoses regarding some items. A smaller proportion of the women with BC reported that they had experienced that healthcare professionals “allowed me to take own responsibility” (OR 0.6; 95% CI 0.4–0.8), “encouraged me to carry through my own solutions” (OR 0.5; 95% CI 0.4–0.7), “made reasonably high demands” (OR 0.6; 95% CI 0.4–0.9), or “sided with me/stood by my side” (OR 0.6; 95% CI 0.4–0.8) compared with women with other SA diagnoses.

**Table 2:** The number and percentages of women on long-term sickness absence (SA) due to breast cancer (BC) (*n* = 187) and other SA diagnoses (*n* = 6010), and odds ratios (OR) with 95% confidence intervals (CI) comparing the different types of encounters experienced among women with BC and women with other SA diagnoses

	Women on SA due to BC n (%)	Women on SA due to other diagnoses n (%)	Crude OR (95% CI) BC compared to other SA diagnoses	Adjusted OR^a^ (95% CI) BC compared to other SA diagnoses
Any positive encounter	179 (95.2)	5716 (95.1)	1.2 (0.6–2.4)	1.1 (0.5–2.2)
Any negative encounter	35 (18.7)	1258 (23.7)	0.7 (0.5–1.1)	0.8 (0.6–1.2)
Believed in my capacity to work	144 (77.0)	4920 (81.7)	0.7 (0.5–1.1)	0.7 (0.5–1.0)
Believed what I said	163 (87.2)	5423 (90.2)	0.7 (0.5–1.1)	0.7 (0.5–1.1)
Respected me	168 (89.8)	5491 (91.4)	0.8 (0.5–1.4)	0.8 (0.5–1.3)
Listened to me	169 (90.4)	5482 (91.2)	0.9 (0.6–1.5)	0.9 (0.5–1.4)
Showed engagement in my case	159 (85.0)	5301 (88.2)	0.8 (0.5–1.2)	0.7 (0.5–1.1)
Allowed me to take own responsibility	144 (77.0)	5149 (85.7)	0.6 (0.4–0.8)	0.6 (0.4–0.8)
Encouraged me to carry through my own solutions	130 (69.5)	4918 (81.8)	0.5 (0.4–0.7)	0.5 (0.4–0.7)
Supported/encouraged me in other ways	144 (77.0)	4876 (81.1)	0.8 (0.6–1.1)	0.8 (0.6–1.1)
Provided adequate and correct information/advice	161 (86.1)	5193 (86.4)	1.0 (0.6–1.5)	1.0 (0.6–1.5)
Was easy to get an appointment with	142 (75.9)	4569 (76.0)	1.0 (0.7–1.4)	1.0 (0.7–1.4)
Took time with me during our meetings	166 (88.8)	5246 (87.3)	1.2 (0.7–1.8)	1.1 (0.7–1.8)
Answered my questions	168 (89.4)	5373 (89.4)	1.0 (0.6–1.7)	1.0 (0.6–1.7)
Made reasonably high demands	149 (79.7)	5197 (86.5)	0.6 (0.4–0.9)	0.6 (0.4–0.9)
Proved to be knowledgeable/competent	169 (90.4)	5389 (89.7)	1.0 (0.7–1.8)	1.1 (0.7–1.8)
Did something beyond what I expected	119 (63.6)	3969 (66.0)	0.9 (0.7–1.2)	0.9 (0.7–1.2)
Was nice to me	169 (90.4)	5482 (91.2)	0.9 (0.6–1.5)	0.9 (0.5–1.5)
Sided with me/stood on my side	138 (73.8)	4999 (83.2)	0.6 (0.4–0.8)	0.6 (0.4–0.8)
Talked about her/himself	61 (32.6)	2282 (38.0)	0.8 (0.6–1.1)	0.8 (0.6–1.1)
Showed that she/he liked me	136 (72.7)	4312 (71.8)	1.1 (0.8–1.5)	1.1 (0.8–1.5)

^a^Adjusted for age (19–44, 45–54, 55–65), country of birth (Sweden, elsewhere), educational level (primary/secondary school, college/university), and depressive symptoms (yes, no)

### Encounters with different healthcare professions

Many of the women had encountered several different types of healthcare professionals during the SA spell and the absolute majority had experienced positive encounters with them. The most frequently mentioned profession was physicians, and in proportionally falling order: registered nurses, physiotherapists, clinical social workers/psychologists, occupational therapists, and naprapaths/chiropractors (data not shown). On the question about whether their contacts with different professions most often were positive or negative, women with SA due to BC and due to other diagnoses, respectively, stated that contacts were most often positive with physicians (BC 95.0%, other 91.3%, *p* = 0.08) and with registered nurses (BC 98.3%, other 95.0%, *p* = 0.04). Similar proportions in both groups of women were observed regarding the other professions (data not shown).

### Encounters’ influence on return to work

**Fig. 1:**
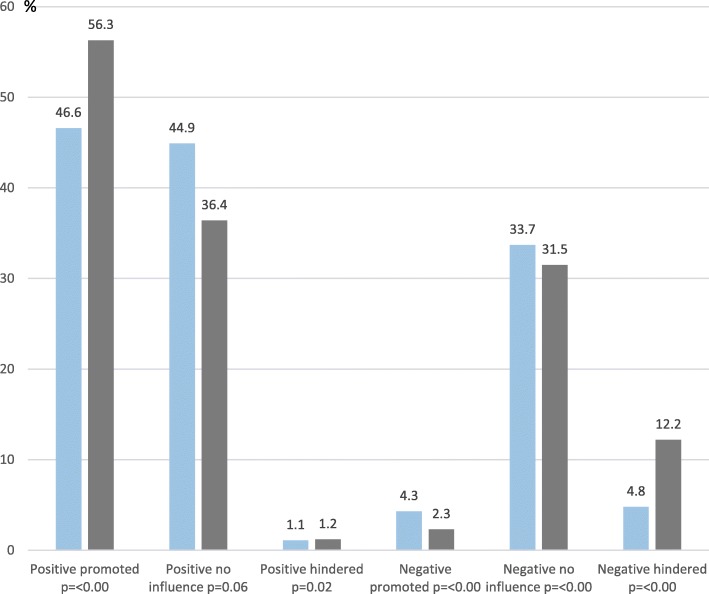
Percentages of women on long-term sickness absence (SA) due to breast cancer (BC) (*n* = 187) and due to other SA diagnoses (*n* = 6010) who had experienced any positive (BC *n* = 173, other *n* = 5645) or negative (BC *n* = 80, other *n* = 2760) encounters from healthcare professionals and stated that those encounters promoted, had no influence, or hindered their ability to return to work, respectively. Women with BC - in blue, women with other SA diagnosis - in grey

About half of the women in both groups reported that positive encounters had promoted their ability to RTW, although in women with SA due to BC the proportion were lower than among women with other diagnoses (BC 46.6%, other 56.3%, *p* < 0.00), while a very small proportion reported that it had hindered RTW (BC 1.1%, other 1.2%, *p* = 0.02) (Fig. 1). When it comes to negative encounters, a small proportion in both groups stated that negative encounters either promoted (BC 4.3%, other 2.3%, *p* < 0.00) or hindered (BC 4.8%, other 12.2%, *p* < 0.00) their ability to RTW. In both groups, about a third stated that positive encounters (BC 44.9%, other 36.4%, *p* = 0.06) or negative encounters (BC 33.7%, other 31.5%, *p* < 0.00) had had no influence on their ability to RTW.

In mutually adjusted multivariable analyses of associations between background variables and having experienced that positive encounters had promoted the ability to RTW, it was observed that: among women on SA due to BC, associations between background variables and positive encounters promoting the ability to RTW were not significant (Table 3). Among women with other SA diagnoses, there was a lower likelihood that positive encounters had promoted the RTW in the oldest age group, 55–65 years old, compared to younger ages, i.e., those who were 19–54 years old (OR 0.7; 95% CI 0.6–0.8), among those not born in Sweden (OR 0.6; 95% CI 0.5–0.7), as well as among those with depressive symptoms (OR 0.8; 95% CI 0.7–0.9). Among those with college/university education there was a higher likelihood that positive encounters had promoted their ability to RTW compared to among those with lower educational level (OR 1.7; 95% CI 1.5–1.8).

**Table 3:** Odds ratios (OR) with 95% confidence intervals (CI) showing the associations between background variables and positive encounters promoting ability to return to work among women on long-term sickness absence (SA) due to breast cancer (BC) (*n* = 187) and among women on long-term SA due to other diagnosis (*n* = 6010), respectively

	Crude OR (95% CI) Women on SA due to BC	Adjusted OR^a^ (95% CI) Women on SA due to BC	Crude OR (95% CI) Women on SA due to other diagnoses	Adjusted OR^a^ (95% CI) Women on SA due to other diagnoses
Age				
19–54	1 (ref)	1 (ref)	1 (ref)	1 (ref)
55–65	0.7 (0.4–1.0)	0.5 (0.3–1.0)	0.8 (0.8–0.9)	0.7 (0.6–0.8)
Country of birth				
Sweden	1 (ref)	1 (ref)	1 (ref)	1 (ref)
Elsewhere	0.5 (0.2–1.2)	0.5 (0.2–1.1)	0.6 (0.5–0.7)	0.6 (0.5–0.7)
Education				
Primary/secondary school	1 (ref)	1 (ref)	1 (ref)	1 (ref)
College/university	1.3 (0.7–2.3)	1.2 (0.7–2.2)	1.7 (1.5–1.9)	1.7 (1.5–1.8)
Depressive symptoms				
No	1 (ref)	1 (ref)	1 (ref)	1 (ref)
Yes	0.9 (0.5–1.9)	1.1 (0.6–2.3)	0.8 (0.7–0.9)	0.8 (0.7–0.9)

^a^Mutually adjusted

Regarding specific types of positive encounters, both among women with BC and women with other diagnoses the statement: having experienced the specific types of positive encounters was associated with having experienced that positive encounters had promoted ability to RTW (Table 4). In sensitivity analyses, the associations did not change when comparing women with BC with the group with all other SA diagnoses excluding other cancer diagnoses (data not shown).

**Table 4:** Odds ratios (OR) with 95% confidence intervals (CI), of women on long-term sickness absence (SA) due to breast cancer (BC) (*n* = 187) and due to other SA diagnoses (*n* = 6010) both stating that they had experienced specific types of positive encounters from healthcare professionals and that positive encounters had promoted their ability to return to work, compared to those who had not experienced such encounters. Separately for BC and other SA diagnoses

	Women on SA due to BC Crude OR (95% CI)	Women on SA due to BC Adjusted OR^a^ (95% CI)	Women on SA due to other diagnoses Crude OR (95% CI)	Women on SA due to other diagnoses Adjusted OR^a^ (95% CI)
Believed in my capacity to work	2.4 (1.2–5.1)	2.4 (1.1–5.1)	3.6 (3.1–4.1)	3.3 (2.9–3.8)
Believed what I said	3.0 (1.1–7.8)	2.8 (1.0–7.5)	3.4 (2.8–4.1)	3.2 (2.6–3.8)
Respected me	5.3 (1.5–19.0)	5.6 (1.5–20.2)	3.7 (3.0–4.5)	3.5 (2.9–4.3)
Listened to me	3.4 (1.1–10.7)	3.7 (1.1–12.0)	3.7 (3.0–4.5)	3.5 (2.9–4.3)
Showed engagement in my case	2.0 (0.9–4.8)	2.1 (0.9–5.0)	3.5 (3.0–4.1)	3.7 (2.8–4.0)
Allowed me to take own responsibility	2.7 (1.1–4.4)	2.0 (1.0–4.2)	3.4 (2.9–4.0)	3.1 (2.7–3.7)
Encouraged me to carry through my own solutions	2.7 (1.4–5.3)	2.6 (1.3–5.2)	3.6 (3.1–4.1)	3.4 (2.9–3.9)
Supported/encouraged me in other ways	2.8 (1.3–5.9)	2.7 (1.3–5.8)	3.3 (2.8–3.7)	3.1 (2.7–3.6)
Provided adequate and correct information/advise	2.7 (1.1–6.7)	2.6 (1.0–6.7)	3.7 (3.1–4.3)	3.5 (3.0–4.2)
Was easy to get an appointment with	3.1 (1.5–6.4)	3.1 (1.5–6.7)	2.2 (1.9–2.4)	2.2 (2.0–2-5)
Took time with me during our meetings	3.1 (1.1–8.9)	3.3 (1.1–9.8)	3.1 (2.6–3.6)	3.0 (2.5–3.5)
Answered my questions	3.7 (1.2–11.5)	3.9 (1.2–12.6)	3.5 (2.9–4.1)	3.3 (2.8–4.0)
Made reasonably high demands	2.6 (1.2–5.5)	2.6 (1.2–5.7)	3.6 (3.1–4.3)	3.4 (2.9–4.0)
Proved to be knowledgeable/competent	4.9 (1.4–17.7)	5.0 (1.4–18.2)	4.0 (3.3–4.8)	3.8 (3.1–4.5)
Did something beyond what I expected	2.5 (1.4–4.7)	2.6 (1.4–5.0)	2.5 (2.3–2.8)	2.5 (2.2–2.8)
Was nice to me	4.9 (1.4–17.7)	5.2 (1.4–19.0)	3.7 (3.0–4.5)	3.6 (2.9–4.3)
Sided with me/stood on my side	2.8 (1.4–5.7)	2.7 (1.3–5.4)	3.0 (2.6–3.4)	2.8 (2.5–3.3)
Talked about her/himself	1.2 (0.6–2.2)	1.1 (0.6–2.0)	1.6 (1.5–1.8)	1.6 (1.4–1.8)
Showed that she/he liked me	2.1 (1.1–4.2)	2.1 (1.0–4.1)	2.3 (2.1–2.9)	2.3 (2.1–2.6)

^a^Adjusted for age (19–44, 45–54, 55–65), country of birth (Sweden, elsewhere), educational level (primary/secondary school, college/university), and depressive symptoms (yes, no)

## Discussion

In this study, exploring how women on long-term SA due to BC or due to other diagnoses, respectively, had experienced their encounters with healthcare professionals, we observed that both groups had mainly experienced positive encounters with healthcare. However, a significantly smaller proportion among women with BC than among those with other diagnoses reported that they had experienced the following four types of positive encounters; “allowed me to take own responsibility”, “encouraged me to carry through my own solutions”, “made reasonably high demands”, and “sided with me/stood by my side”. About half of all the women stated that encounters with healthcare professionals had influenced their ability to RTW, however, this proportion was smaller among the women on SA with BC. There were differences between the two groups of women regarding age, and depressive symptoms, reflecting that mental SA diagnoses were common in the group with other SA diagnoses. Further, a lower proportion among women who were older, not born in Sweden, had lower educational level, or depressive symptoms experienced that positive encounters promoted their ability to RTW. These results can be used as basis for intervention programs in oncology clinics and in other healthcare settings caring for these women.

Both groups of women reported that their encounters with healthcare professionals during their long-term SA in general were positive, which is in line with the results from previous studies [18, 19, 24, 41]. Moreover, a smaller proportion of the women with BC reported having experienced negative encounters compared to the women with other SA diagnoses, and more research is needed to elucidate the reasons for this difference. Even if a very high proportion of the women in both groups in this study stated that they had experienced positive encounters, the question may be raised if healthcare professionals act differently when the patient has been diagnosed with cancer compared to other diagnoses. Are patients with diagnoses that to a higher extent are based on objective measures, encountered in a more positive way compared to those with e.g., musculoskeletal pain or mental disorders, where both diagnosis and etiology can be unclear or difficult to verify objectively [42]?

Regarding specific types of experienced positive encounters, 77.3% of the women with BC reported that they had experienced that healthcare professionals believed in their capacity to work. A slightly larger proportion of those with other SA diagnoses (81.9%) reported having experienced this encounter. In several studies, physicians report that regarding sickness certification of patients, it is problematic to assess patients work capacity, however, oncologist report this to a lesser extent than other physicians [43–45].

Other differences observed between the groups of women in this study were that a smaller proportion of women with BC compared with women with other SA diagnoses experienced that healthcare professionals “allowed me to take own responsibility” or “encouraged me to carry through my own solutions”. An important aspect to facilitate RTW has been shown to be possibilities for adjustments and flexibility, regarding work as well as healthcare and treatment [33, 46, 47]. The medical aspects of BC treatment are, however, rather standardized. Since adjustments and flexibility in connection to healthcare requires that the patient is allowed to take action, and to find solutions, this may indicate an area where women on SA with BC are somewhat prevented from experiencing such types of encounters from healthcare. Further research regarding this is important.

In both groups, we found that there were associations between having experienced specific types of positive encounters and whether positive encounters were experienced as having promoted the women’s ability to RTW. This is in line with findings from previous studies [11, 26–28, 32, 33]. Examples of such specific encounters are that healthcare professionals provide adequate and correct information/advice or answer questions. The importance of information has also been shown in previous studies, e.g., that informing women with BC about side-effects of treatments and how those can influence work capacity, promotes RTW [31, 33]. Other positive types of encounters in our study were: “respected me”,“ encouraged me to carry through own solutions”, and “supported/encouraged me in other ways” which also have been shown to promote RTW among sickness absentees in general [19].

In both groups, a large number of women indicated that neither positive nor negative encounters had any influence on their ability to RTW. Of course, RTW could be influenced by several factors, operating at many different structural levels [8, 11, 13]. It could also be hypothesized that the positive encounters experienced during the SA were not tailored to the women’s needs and consequently had no influence on their RTW, or that RTW was not an option at all, given the patient’s condition.

A major focus for healthcare in relation to BC, is to diagnose BC at an early phase, to treat the BC, and to follow the effects of treatment for the patient. Nevertheless, more than half of the participants stated that healthcare encounters had impacted on their ability to RTW, mainly so that positive encounters promoted and negative hindered RTW. That the encounters from healthcare professionals have an impact on RTW to such a large extent as shown in this study is in line with previous research [16–24].

A larger proportion of the oldest women in the group with other SA diagnoses stated that the encounters had not promoted their ability to RTW. This could be related to the fact that encounters related to work issues and SA may be influenced by how established one is on the labor market or by the expected remaining time of working life. In our study, a smaller proportion of those with other SA diagnoses who were not born in Sweden stated that positive encounters promoted their ability to RTW; this was not the case for women on SA due to BC. This finding is not in line with a previous literature review regarding RTW showing that ethnicity may influence RTW among women with BC [7]. This discrepancy may be because our questionnaire was only available in Swedish, resulting in a higher selection of women established in the Swedish society. We also found an association between having college or university education and having experienced that positive encounters promoted the ability to RTW among the women with other SA diagnoses – however, not in the BC group. This may be due to group size as well as the possibly larger heterogeneity within the group of women with other SA diagnoses compared to within the group of women with BC (who in general had a higher educational level).

Depression may hinder RTW per se but also influence how women experience different encounters [7, 37] which is why we included two questions to assess self-rated depressive symptoms, in the analyses. However, among the women with BC, we did not detect a statistically significant association between depressive symptoms and experiencing that positive encounters promoted the ability to RTW, which may be due to the smaller number of women in this group. On the other hand, among the women with other SA diagnoses, a larger proportion reported depressive symptoms, which might reflect that the most frequent SA diagnosis in this group was a mental diagnosis, but also that such symptoms might have influenced their perception that positive encounters had promoted their ability to RTW.

In this study we aimed to gain knowledge of whether the experiences of women on long-term SA with BC differed from those of women on long-term SA due to other diagnoses. Differences between the two groups in terms of age, birth country, and other sociodemographic factors were handled through statistical adjustments. To further explore these results, longitudinal studies are needed, and also that intervention studies, with the aim to develop healthcare professionals’ competence regarding handling work issues and SA of women with BC, are developed and tested scientifically.

### Strengths and limitations

A strength of the current study is that it is based on a large, randomly selected population-based sample including half of all individuals in Sweden who had an ongoing SA spell that had lasted at least four and not more than eight months. A strength is also that background data from nationwide registers of high quality could be used, enabling both non-response, and sub-group analyses with adjustment for important sociodemographic factors. Other strengths are that the questionnaire is based on previously used questions about types of encounters and their influence on RTW.

An additional strength is that the study is conducted in Sweden, with a very high employment frequency among women, also in higher ages, meaning that the healthy-selection effect of women into the work force is not as strong as in other countries.

Regarding limitations: we do not know how the professionals actually encountered the women. However, the most important influence on ability to RTW would likely be how the woman herself actually experienced the encounters [17]. Another limitation is the response rate of 55.4%. As in most surveys, the response rate was lower among those with lower education, in lower ages, and among those not born in Sweden. The latter may be due to that the questionnaire was only available in Swedish. In general, the proportion of people not born in Sweden is higher among long-term sickness absentees than in the rest of the population [48]. Due to this, and also since many of the participants may have had severe morbidity, with functional limitations making them unable to answer – or sometimes even read the questions – it can be considered a relatively high response rate. Even though we had the possibility to use register data to analyze proportions of non-responders it was not possible to contact them, which means that we do not know if we have a response bias towards those mainly having experienced positive or negative encounters – or no such bias. Nevertheless, the large sample size, of more than six thousand women, provides a wide panorama of experiences of encounters. Furthermore, though we had a large overall sample, the relatively low number of women with BC limited the possibilities for some analyses in this group. In some of the comparisons when significant results were only observed in the group of women with other SA diagnoses and not in the BC group, it may be related to the limited statistical power among women on SA due to BC.

Factors other than encounters that have been shown to be associated with the ability to RTW are disease-, treatment- and work-related factors as well as social support and financial independency [7, 30, 46]. We had no information about such aspects, and thus could not take them into account. However, in the regression analyses when adjusting for age, country of birth, educational level, and depressive symptoms, we in general observed very small changes in the ORs. Nevertheless, additional studies are needed regarding differences within the groups of women and how they have experienced encounters with healthcare related to work and SA, as well as if the experience of encounters varies over time, that is during the SA spell, e.g., regarding disease state and treatment.

## Conclusion

The majority of the women on long-term SA due to BC or due to other SA diagnoses had experienced positive encounters, in general as well as regarding specific types of positive encounters with healthcare professionals, while few had experienced any negative encounter. As many as half of the women stated that the positive encounters they had experienced promoted their ability to RTW, although a slightly smaller proportion of the women with BC stated that. For specific types of positive encounters there were no difference between the two groups, except for the following types of encounters that were experienced by a smaller proportion of the women on SA due to BC: “allowed me to take own responsibility”, “encouraged me to carry through my own solutions”, “made reasonably high demands”, and that the healthcare professional “sided with me/stood on my side”. This study emphasizes that not only medical treatment but also the encounters with the healthcare professionals themselves may influence the ability to RTW, something that is of clinical importance.

## Abbreviations

BC: Breast cancer
RTW: Return to work
SA: Sickness absence
